# Use of Nebulized Hypertonic Saline in Patients With Neuromuscular Diseases or Cerebral Palsy in the United Kingdom

**DOI:** 10.1002/ppul.27464

**Published:** 2024-12-31

**Authors:** Natalia Galaz‐Souza, Hui‐Leng Tan, Matthew Hurley, Andrew Bush

**Affiliations:** ^1^ Imperial College London, National Heart and Lung Institute London UK; ^2^ Nottingham University Hospitals NHS Trust Nottingham UK; ^3^ Department of Paediatric Respiratory Medicine Royal Brompton and Harefield Hospitals London UK; ^4^ Division of Child Health University of Nottingham Nottingham UK

To the Editor,

## 1 Introduction

People with neuromuscular diseases (NMDs) and complex neurodisability (such as cerebral palsy, CP) have significant respiratory morbidity, mainly related to reduced ventilatory function and cough efficacy, and incoordinate swallowing and inadequate airway protection. Muscle weakness may lead to ineffective coughing and secretion retention [1]. Additionally, impaired laryngeal sensation, gastroesophageal reflux, and silent aspiration can result in atelectasis [2], and changes in chest wall mechanics due to scoliosis all put the respiratory muscles at a mechanical disadvantage. While there is no evidence of primary mucociliary impairment or abnormal sputum rheology in these patients, recurrent infections can secondarily impair mucociliary clearance. Respiratory exacerbations are the leading causes of emergency admissions, hospitalizations, and death in NMD patients [3], negatively impacting quality of life. Nebulized hypertonic saline (HS) assists airway clearance by reducing mucus viscosity and hydrating the airway surface liquid. Most evidence is in cystic fibrosis (CF), wherein nebulized 7% HS reduces exacerbations and improves quality of life [4]. There is only retrospective observational evidence of benefit in NMDs and neurodisability [5].

## 2 Methods

We conducted a survey to audit current prescription practices for nebulized HS in NMDs and CP patients. As an audit, research ethics committee approval was not required. The survey ran from 23‐05‐2023 to 31‐12‐2023. We used the Qualtrics online platform to create and distribute the survey to healthcare professionals involved in respiratory care for NMD and CP patients. Distribution channels included work and academic emails, anonymous links, and QR codes shared through professional networks (The Association of Chartered Physiotherapists in Respiratory Care and British Thoracic Society), social media (ResearchGate, LinkedIn, X, and Facebook), and the British Thoracic Society Winter Meeting 2023. The survey employed multiple‐choice questions and free text entries. Qualitative data from free text entries was analyzed using a framework method. Categories were developed using a mixed method (i.e., deductive and inductive). The framework categorizes cases by region in the United Kingdom and then by center. The topics included were (1) HS versus NS; (2) When to use nebulized HS in NMD and CP; (3) Why would you not use nebulized HS in NMD and CP; (4) Choice of concentration of HS; (5) Daily versus as needed (PRN) use; (6) Assessment of effectiveness; (7) Safety considerations; (8) Side effects observed; (9) Use in NMD versus CP; (10) Need for/lack of evidence/guidelines; (11) Other. Coding and analysis were performed using the NVIVO 14 software.

## 3 Results

We received 66 responses from the 12 regions in the United Kingdom, most from the East Midlands (*n* = 15, 22.8%), London (*n* = 10, 15.2%), and South West (*n* = 10, 15.2%). There were 55 responses from tertiary centers, one from secondary center, four from community health, one from private practice, and five from unknown centers. Respondents were predominantly physiotherapists (*n* = 35, 53%; 33 from pediatric services), followed by doctors (*n* = 28, 42.4%; 27 pediatricians) and clinical nurse specialists (*n* = 3, 4.5%).

**Figure 1:**
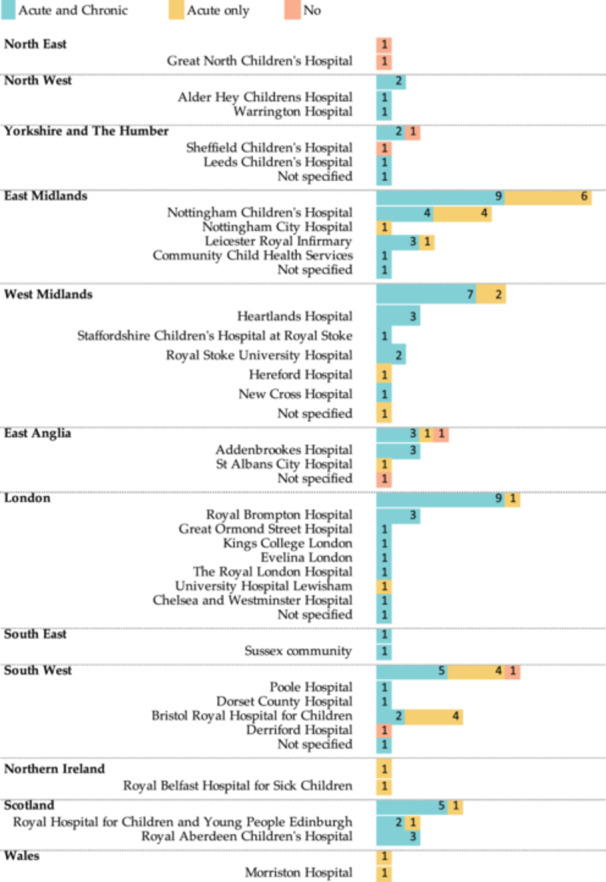
Bar chart of survey responses according to the 12 UK regions, subdivided by center. North East (*n* = 2, Centers = 2), North West (*n* = 2, Centers = 2), Yorkshire and The Humber (*n* = 3, Centers = 3), East Midlands (*n* = 15, Centers = 5), West Midlands (*n* = 9, Centers = 6), East Anglia (*n* = 5, Centers = 3), London (*n* = 10, Centers = 8), South East (*n* = 1, Centers = 1), South West (*n* = 10, Centers = 5), Northern Ireland (*n* = 1, Centers = 1), Scotland (*n* = 6, Centers = 2), Wales (*n* = 1, Centers = 1).

Nebulized HS was prescribed or recommended by 93.9% (*n *= 62) of respondents for pulmonary exacerbations (30.3%, *n *= 20) and chronic therapy (63.3%, *n *= 42). Four respondents did not use nebulized HS due to a lack of evidence of efficacy (*n *= 3) and treatment burden (*n *= 1). There were regional differences in prescribing patterns across the United Kingdom. For example, in the East Midlands, over half of the respondents (60%%) used nebulized HS both during pulmonary exacerbations and as chronic therapy, whereas in London, the vast majority used it for acute and chronic therapy (90%). There were also and differences within centers. For example, in Nottingham Children's Hospital, half of the respondents used nebulized HS both during pulmonary exacerbations and as chronic therapy, while half used it only during acute exacerbations (Figure 1).

Several free‐text entries commented that nebulized HS was useful and mostly well‐tolerated, especially in complex neurodisability, and that it helped empower families in the management of their children.

**Figure 2:**
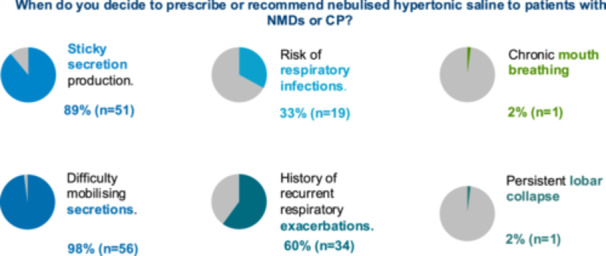
Pie charts of main indications to prescribe or recommend nebulized HS to patients with NMDs or CP. Sticky secretion production (*n* = 51, 89%); Difficulty mobilizing secretions (*n* = 56, 98%); Risk of respiratory infections (*n* = 19, 33%); History of recurrent respiratory exacerbations (*n* = 34, 60%); Other (*n* = 2, 4%).

The main indication for HS was difficulty mobilizing secretions (98%, *n *= 56) and sticky secretions (89%, *n *= 51). Other indications included recurrent pulmonary exacerbations (60%, *n *= 34); when there is a risk of respiratory infections (33%, *n *= 19); chronic mouth breathing (2%, *n *= 1); and persistent lobar collapse (2%, *n *= 1) (Figure 2). The main reasons for not using nebulized HS in this population were the lack of evidence of efficacy and the treatment burden.

### 3.1 HS Versus NS

When deciding between normal saline (NS) or HS, most clinicians considered the severity of the symptoms (77%, *n* = 50) and previous response to the treatment (69%, *n* = 45). Three respondents used HS in escalation plans. Other criteria included the clinician's previous experience (52%, *n* = 34), the physiotherapist's advice (*n* = 6), the availability of treatment (*n* = 3), and the MDT discussion (*n* = 1).

### 3.2 Choice of Concentration of HS

Tolerability (68%, *n* = 40) and previous response (61%, *n* = 36), clinical presentation (56%, *n* = 33), the clinician's previous experience (53%, *n* = 31), the availability of the treatment (10%, *n* = 6), patient's age (7%, *n* = 4), physiotherapist's advice (5%, *n* = 3), thickness of secretions (2%, *n* = 1), and trial and error (2%, *n* = 1) were the main criteria for choice of concentration. Six respondents escalated from 3%, while only two stepped down from 7% if this was not tolerated.

### 3.3 Daily Versus as PRN

Most decided whether to prescribe HS daily or PRN depending on the patient's clinical presentation (79%, *n* = 49) and previous response to the treatment (63%, *n* = 39). Others used frequency of symptoms or exacerbations (2%, *n* = 1), clinician's previous experience (48%, *n* = 30), and the physiotherapist's advice (6%, *n* = 4) to decide.

### 3.4 Side Effects

The most common side effects observed by respondents were wheezing (53%, *n* = 33) and intense coughing (34%, *n* = 21). Excessive secretion production was reported by 24% (*n* = 15). Other side effects reported included sore throat (*n* = 5), vomiting (*n* = 1), general discomfort (*n* = 1), discomfort related to mask (*n* = 1) and aftertaste (*n* = 1). A total of 23% (*n* = 14) had observed no side effects.

### 3.5 Assessment of Effectiveness

Effectiveness was assessed by improvement in respiratory symptoms (89%, *n* = 58), reduced exacerbation frequency (65%, *n* = 42), and quality of life (54%, *n* = 35). Only seven used pulmonary function tests. Other means to assess effectiveness included need for respiratory support (*n* = 2), carer's opinion (2%, *n* = 1), and reduction in suctioning (*n* = 1).

### 3.6 Safety Considerations

Four respondents reported that they addressed potential bronchoconstriction by doing a drug response assessment or prescribing a predose of bronchodilator.

### 3.7 Use in NMD Versus CP

Concerns were expressed about using HS in NMDs (but not in neurodisability), mainly regarding weak cough. Sixteen respondents were concerned that HS could cause excessive secretions that would be difficult to clear, especially in NMDs. Three respondents reported that they prescribe HS only after a detailed evaluation of cough strength and if used as an adjunct to other airway clearance techniques. One respondent refrained from using in NMD, but used in CP.

### 3.8 Other

Another theme was the importance of the community teams' involvement in supporting the extra equipment for HS and their confidence in continuing treatment in the community.

### 3.9 Need for/Lack of Evidence/Guidelines

Seven respondents shared the need for evidence, and one was concerned that nebulized HS might be overused in this population.

## 4 Discussion

Respiratory morbidity is a significant issue in people with NMDs and neurodisability. There is preliminary retrospective evidence suggesting the potential benefit of nebulized HS to reduce respiratory morbidity in NMD and neurodisability. However, no prospective studies have assessed effectiveness and safety in this group, and its use is currently off‐label.

Our findings show that, although there is no published evidence of efficacy in NMDs and CP, a high percentage of centers in the United Kingdom use nebulized HS both during acute exacerbations and as chronic treatment in this population.

These results offer the first information about current practices for nebulized HS in NMDs and CP. The findings provide insight and shed light on the heterogeneity of current practices, highlighting the need for research. There is a clear need for well‐designed studies to establish efficacy, optimize dosing, and develop guidelines tailored to these patient populations.

Concerns about HS usage in NMDs due to weak cough and the risk of excessive secretions was mentioned in the update on standards of care recommendations for SMA [6] and highlighted the necessity for careful patient evaluation and personalized treatment plans. Additionally, the importance of community team support for continuing HS treatment at home is evident.

## 5 Conclusions

In conclusion, this survey shows that, despite the scarcity of evidence, a significant percentage of clinicians prescribe HS both during acute exacerbations and as chronic treatment. However, there is significant variation in practice across the United Kingdom, emphasizing the need for clinical trials to establish evidence‐based guidelines.

## Author Contributions

**Natalia Galaz‐Souza:** conceptualization, data curation, formal analysis, investigation, methodology, visualization, writing of the original draft. **Hui‐Leng Tan:** supervision, writing review and editing. **Matthew Hurley:** supervision, writing review and editing. **Andrew Bush:** supervision, writing review and editing.

## Conflicts of Interest

The authors declare no conflicts of interest.

## Data Availability

The data that support the findings of this study are available from the corresponding author upon reasonable request.
